# P-1092. Re-analysis of Pivmecillinam Randomized Clinical Trial Data According to 2019 US Food and Drug Administration Guidance for the Treatment of Uncomplicated Urinary Tract Infection

**DOI:** 10.1093/ofid/ofae631.1280

**Published:** 2025-01-29

**Authors:** Keith S Kaye, Anita F Das, Niels Frimodt-Møller, Kalpana Gupta, Thomas Lodise, Anne Santerre Henriksen, Morton Alexander, Florian Wagenlehner

**Affiliations:** Rutgers Robert Wood Johnson Medical School, New Brunswick, NJ; Das Consulting, Guerneville, CA; Rigshospitalet, Copenhagen, Hovedstaden, Denmark; VA Boston, Boston, MA, USA, Boston, Massachusetts; Albany College of Pharmacy and Health Sciences, Albany, New York; Utility Therapeutics, Jyllinge, Hovedstaden, Denmark; Utility Therapeutics, Jyllinge, Hovedstaden, Denmark; Justuf Liebeg University Diessen, Diessen, Hessen, Germany

## Abstract

**Background:**

Due to increasing prevalence of resistant uropathogens, there is urgent need in the USA for effective new oral antibiotics for treatment of uncomplicated urinary tract infection (uUTI). Pivmecillinam has been used to treat uUTI in Canada and Europe for > 40 years, is recommended as a first-line agent by the Infectious Diseases Society of America, and has high microbiologic activity against most antibiotic-resistant Enterobacterales. We present a re-analysis of clinical trial data, as per 2019 US Food and Drug Administration (FDA) guidance, that supported the recent US approval of pivmecillinam for treatment of uUTI.

**Table 1.**
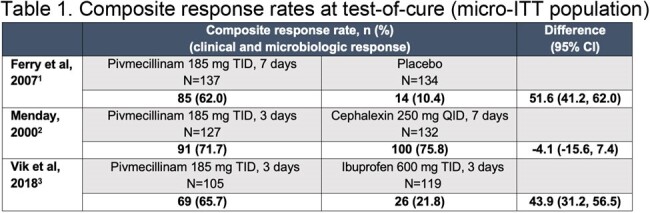
Composite response rates at test-of-cure (micro-ITT population) 185 mg of pivmecillinam is equivalent to 200 mg of pivmecillinam hydrochloride. CI, confidence interval; micro-ITT, microbiological intent-to-treat; QID, four times daily; TID, three times daily. 1. Ferry SA et al. Scand J Prim Health Care. 2007;25:49–57. 2. Menday AP. Intl J Antimicrob Agents. 2000;13:183–187. 3. Vik I et al. PLoS Med. 2018;15:e1002569.

**Methods:**

Detailed subject-level clinical data were retrieved from three historic randomized controlled trials (RCTs) including subjects treated with pivmecillinam 185 mg, three times daily for 3–7 days, and re-analyzed in accordance with the 2019 FDA guidance for uUTI. Efficacy endpoints were composite (clinical and microbiologic) response rates, clinical response rates, and microbiologic response rates, analyzed in the microbiological intent-to-treat population (urine culture ≥ 10^5^ colony-forming units/mL; ≤ 2 microorganism species; no baseline pathogen non-susceptible to active comparator).

**Table 2.**
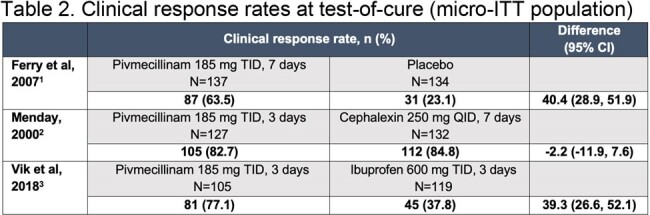
Clinical response rates at test-of-cure (micro-ITT population) 185 mg of pivmecillinam is equivalent to 200 mg of pivmecillinam hydrochloride. CI, confidence interval; micro-ITT, microbiological intent-to-treat; QID, four times daily; TID, three times daily.

1. Ferry SA et al. Scand J Prim Health Care. 2007;25:49–57. 2. Menday AP. Intl J Antimicrob Agents. 2000;13:183–187. 3. Vik I et al. PLoS Med. 2018;15:e1002569.

**Results:**

Efficacy in uUTI was demonstrated for the recommended dosage regimen of pivmecillinam in the three studies (total N=369 pivmecillinam, N=385 comparators). Composite response rates for pivmecillinam ranged from 62.0% to 71.7% (**Table 1**), clinical response rates from 63.5% to 82.7% (**Table 2**), and microbiologic response rates from 74.3% to 86.9% (**Table 3**). Efficacy of pivmecillinam was superior to placebo or ibuprofen and similar to cephalexin. Clinical and microbiologic response rates for pivmecillinam and comparators were generally numerically lower in the re-analysis versus original reports, reflecting the more stringent 2019 FDA criteria.

**Table 3.**
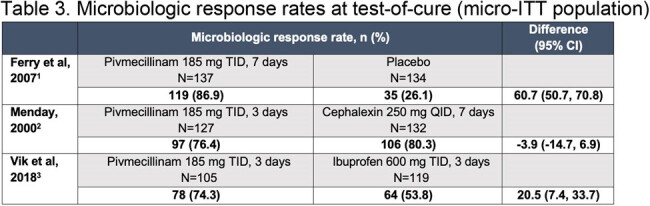
Microbiologic response rates at test-of-cure (micro-ITT population) 185 mg of pivmecillinam is equivalent to 200 mg of pivmecillinam hydrochloride. CI, confidence interval; micro-ITT, microbiological intent-to-treat; QID, four times daily; TID, three times daily.

1. Ferry SA et al. Scand J Prim Health Care. 2007;25:49–57. 2. Menday AP. Intl J Antimicrob Agents. 2000;13:183–187. 3. Vik I et al. PLoS Med. 2018;15:e1002569.

**Conclusion:**

This re-analysis of RCT data confirmed the efficacy of pivmecillinam in uUTI, and was used to support the recent US approval of oral pivmecillinam, 185 mg three times daily for 3–7 days, for the treatment of female patients aged ≥ 18 years with uUTI caused by susceptible isolates of *Escherichia coli*, *Proteus mirabilis,* and *Staphylococcus saprophyticus*.

**Disclosures:**

**Keith S. Kaye, MD, MPH**, Allecra: Advisor/Consultant|CARB-X: Advisor/Consultant|GSK: Advisor/Consultant|Merck: Advisor/Consultant|Shionogi: Advisor/Consultant|Spero: Advisor/Consultant **Anita F. Das, PhD**, Cidara: Advisor/Consultant|Contrafect: Advisor/Consultant|Iterum Therapeutics: Advisor/Consultant|Paratek: Advisor/Consultant|Utility therapeutics: Advisor/Consultant **Niels Frimodt-Møller, MD DMSc**, Eli Lilly Ltd: Stocks/Bonds (Private Company)|Pfizer Ltd: Stocks/Bonds (Private Company)|Rosco A/S: Advisor/Consultant **Kalpana Gupta, MD**, GlaxoSmithKline: Advisor/Consultant|IDSA GL on UTI: Uncompensated author|Iterum Therapeutics: Advisor/Consultant|PhenUtest Diagnostics: Advisor/Consultant|Qiagen Inc.,: Advisor/Consultant|UpToDate: Royalties for UTI topics|Utility Therapeutics Ltd: Advisor/Consultant **Thomas Lodise, Jr., Pharm.D., PhD**, MERCK: Advisor/Consultant **Anne Santerre Henriksen, PhD**, Utility therapeutics: Advisor/Consultant **Morton Alexander, PhD**, SNIPR biome: Shareholder|SNIPR biome: Stocks/Bonds (Private Company)|Union therapeutics: Shareholder|Union therapeutics: Stocks/Bonds (Private Company)|Utility therapeutics: Board Member|Utility therapeutics: Ownership Interest **Florian Wagenlehner, MD**, Astellas: Advisor/Consultant|AstraZeneca: Advisor/Consultant|Bionorica: Advisor/Consultant|DFG (German Research Foundation) funded research group BARICADE (FOR5427/1-466687329): Speaker|GSK: Advisor/Consultant|GSK: Principal investigator in a GSK-sponsored study|Janssen: Advisor/Consultant|Klosterfrau: Advisor/Consultant|MIP Pharma: Advisor/Consultant|OM Pharma: Advisor/Consultant|Spero: Advisor/Consultant|VenatoRX: Advisor/Consultant

